# Baduanjin exercise for cervical spondylotic radiculopathy: A protocol for systematic review and meta-analysis

**DOI:** 10.1097/MD.0000000000020037

**Published:** 2020-05-01

**Authors:** Wenkang Dai, Xiongwei Wang, Rui Xie, Minghui Zhuang, Xiaojuan Chang, Guoqing Yang, Jie Yu, Liguo Zhu

**Affiliations:** a Wangjing Hospital of China Academy of Chinese Medical Sciences; b Beijing University of Chinese Medicine, Chaoyang District, Beijing; c Guizhou University of Traditional Chinese Medicine, Guiyang, China.

**Keywords:** Baduanjin, cervical spondylotic radiculopathy, meta-analysis, protocol

## Abstract

**Background:**

Cervical spondylotic radiculopathy (CSR) is one of the most common public health concerns in the world. Baduanjin is very widely and popularly practiced for the management of CSR. Therefore, we conducted a systematic review and meta-analysis to investigate the efficacy of Baduanjin exercise for patients with CSR.

**Methods:**

The PubMed, Web of Science, Embase, Cochrane Central Register of Controlled Trials, Clinical Trials.gov, Cochrane Library, SinoMed, Chinese National Knowledge Infrastructure Database, Wan Fang database, and VIP databases were searched from inception to July 2019 to identify potentially eligible studies. The methodological quality of the included studies using the risk bias assessment tool of Cochrane. All statistical analysis are conducted with Revman 5.3.

**Results:**

This systematic review and meta-analysis will provide a synthesis of existing evidences for the treatment of Baduanjin on CSR.

**Conclusion:**

The conclusions of this study will provide evidence to evaluate the effectiveness of Baduanjin for CSR, which can further guide the promotion and clinical decisions.

**PROSPERO registration number:**

CRD42020149659

## 1 Introduction

Cervical spondylotic radiculopathy (CSR) is a series of clinical manifestations caused by degenerative changes in nerve root compression.^[1]^ The annual incidence of a CRS is estimated at 0.8 per 1000 inhabitants.^[2]^

Many patients are in their productive phase of life. CSR severely affects patients’ quality of life. Loss of productivity from pain ranging from $ 29.9 billion to $ 335 billion.^[3]^

In recent years, Tai chi, Endurance training, UE stretching or ROM xexercise, Baduanjin, and other sports therapy have become more and more popular among the public for CSR management. A 2015 Cochrane systematic review study concluded that using specific strengthening exercises as a part of routine practice for chronic neck pain and radiculopathy may be beneficial. Research showed the use of strengthening and endurance exercises for the cervico-scapulothoracic and shoulder may be beneficial in reducing pain and improving function.^[4]^ A Baduanjin review study concluded that mindfulness-based Baduanjin exercise may be effective for alleviating musculoskeletal pain and improving overall sleep quality in patients with chronic diseases.^[5]^

A 2017 advances in the diagnosis and management of neck pain form the Johns Hopkins Medical School, Mayo Clinic discussed the potential benefits of yoga, and spinal manipulation for CRS, but no mention of Baduanjin.^[6]^

Baduanjin exercise includes 8 independent movements. Baduanjin is characterized by symmetrical body postures and movements, breathing control, mental concentration, and meditation.^[7]^ Baduanjin strengthens the strength of the neck and back muscles to maintain the stability of the cervical spine. It can relieve muscle spasms, improve bone structure, reduce pain, prevent muscle atrophy, restore and improve cervical spine movement function, prevent cervical joint stiffness, and improve blood circulation in the neck to promote the resolution of inflammation.^[8–12]^ The guidelines do not give a recommendation for Baduanjin treatment of CSR.^[13]^

Therefore, the purpose of this study is to systematically evaluate the clinical efficacy of Baduanjin in treating CSR.

## 2 Methods

### 2.1 Study registration

Since this study is a secondary literature study based on randomized controlled trials (RCTs), no ethical approval is required.

The protocol of this study has been registered on the International Prospective Register of Systematic Reviews (registration no.CRD42020149659, which is available on https://www.crd.york.ac.uk/prospero/display_record.php?ID=CRD42020149659) basing on the preferred reporting items for systematic reviews and meta-analyses protocols statement guidelines.^[14]^

### 2.2 Inclusion criteria for study selection

#### 2.2.1 Type of studies

We will include RCTs and semi-RCTs. Cross-over trials, review, case reports, cohort studies, experimental studies, expert experience, and other non-RCTs will be excluded. There are no restrictions on languages.

#### 2.2.2 Type of participants

We will include studies that patients diagnosed as CRS, not limited by age, sex, race, nationality. Patients should have been diagnosed with CRS based on past or current guidelines for the diagnosis of CRS.

#### 2.2.3 Type of interventions

We will include the studies that Baduanjin alone, or in combination with another active treatment (such as a pharmacologic or non-pharmacologic treatment), while we have no restrictions on intervention in the control group and the control group did not include Baduanjin.

#### 2.2.4 Type of outcome measurements

##### 2.2.4.1 Primary outcomes

The visual analog scale will be defined as the primary outcome for assessing the degree of pain after treatment.

##### 2.2.4.2 Secondary outcomes

The simplified Mc Gill Pain Questionnaire(MPQ) was used to evaluate the degree of pain, including pain score, visual analog scale, and current pain degree; the quality of life; Yasuhisa Tanaka 20 score.

### 2.3 Search methods for the identification of studies

#### 2.3.1 Data sources

Search the following databases from their inception to September 2019: PubMed, Web of Science, EMBASE, Cochrane Central Register of Controlled Trials, ClinicalTrials.gov, Cochrane Library, SinoMed, Chinese National Knowledge Infrastructure Database, Wan Fang database, and VIP databases. No limits were imposed on the dates, types, and statuses of the publications eligible for inclusion. We will also retrieve any other gray literature sources. There will be no limitation on language, publication type, and status.

#### 2.3.2 Search strategy

The key terms used in the searches were: “nerve root cervical spondylotic,” “cervical spondylosis,” “cervical spondylotic radiculopathy,” “cervical spondylopathy,” “cervical syndrome,” “neck pain,” “mechanical neck disorders,” “baduanjin,” “baduan jin,” “ba duan jin,” “eight section brocades,” “brocades,” “eight trigrams boxing,” “eight-treasured exercises,” “eight pieces of brocade,” “qigong.” Different search strategies were used for the Chinese and foreign language databases. When possible, we will contact report authors for additional information. No language or publication status restrictions will be imposed.

### 2.4 Selection of studies

In this study, 2 researchers will independently screen the literature based on the inclusion and exclusion criteria. Articles with inconsistent results from the researcher's screening will be resolved by discussion with the opinion of a third researcher.

### 2.5 Data extraction and management

**Figure 1:**
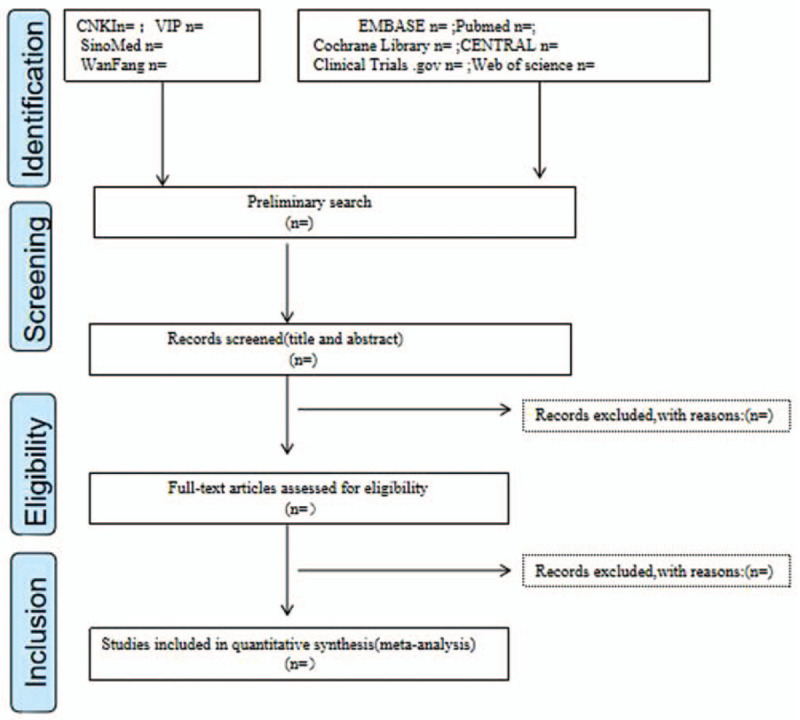
Flow diagram of study selection and screening process.

We will use EndNoteX9 software to manage the retrieved literature. The following information was independently extracted by 2 researchers using a predetermined form: the publication time of the literature, the name of the first author, gender, age, sample size, interventions in the experimental and control groups, outcomes, follow-up duration. When relevant data has not been reported, we will contact the authors by email or in other ways to attempt to obtain the missing information. Disagreements are resolved by consensus or in consultation with a third review author. The preferred reporting items for systematic review and meta-analysis flow diagram (Fig. 1) will be used to show the details of the study selection process.

### 2.6 Assessment of risk of bias

Two of our researchers will use the bias risk tool provided by the Cochrane Collaboration^[15]^ to evaluate the quality of the literature using RevMan 5.3 software (Copenhagen, The Nordic Cochrane Centre, The Cochrane Collaboration, 2014). This recommended tool includes 7 important items: sequence generation, allocation concealment, blinding of participants and personnel, blinding of results evaluation, incomplete result data, selective result reporting, and other biases. Make “Low risk,” “High risk,” and “unclear risk” judgments for each research literature. Finally, a “risk of deviation” summary and a chart are generated to show the results.

As with the previous process, it will be independently assessed by 2 researchers. If there is disagreement, it will be discussed with the 3rd researcher.

### 2.7 Data analysis

This study will use RevMan 5.3 software provided by the Cochrane Collaboration Network for meta-analysis. Dichotomous data will use odds ratio as the effect analysis statistics, and measurement data will use mean difference as the effect analysis statistics, and both will use the effect amount and its 95% confidence interval. If *P* > .1 or *I*^2^ ≤ 50%, we will use a fixed effect model for meta-analysis; if *P* < .1 or *I*^2^ > 50%, we will analyze data using a random-effects model.

### 2.8 Sensitivity analysis

In order to evaluate the sensitivity of the meta-analysis, studies will be conducted to exclude one by one and analyze the source of heterogeneity.

### 2.9 Reporting bias analysis

If there are more than 10 qualified studies are included in our study, funnel plots and Egger regression analysis will be carried out to assess the publication bias. We will discuss the sources and explanations of bias.

## 3 Discussion

CSR is a common disease that causes a huge burden on society and the economy. Recently, Baduanjin is gaining increasing popularity among the public for CRS management.^[4,6,16]^

Baduanjin can strengthen the strength of the neck and back muscles to maintain the stability of the cervical spine, prevent muscle atrophy, restore and improve the movement function of the cervical spine.^[8–12]^ Some researchers have found that exercise can activate conditioned pain to modulate the downward inhibition pathway, leading to subsequent pain relief.^[17]^

This systematic review and meta-analysis study aims to assess the effectiveness and safety of Baduanjin in the treatment of CSR.

Through analysis and integration of existing clinical studies, it can provide a basis for further guiding clinical selection of appropriate interventions.

## Author contributions

**Conceptualization:** Wenkang Dai, Jie Yu.

**Data curation:** Xiongwei Wang, Rui Xie.

**Investigation:** Minghui Zhuang, Xiaojuan Chang.

**Methodology:** Wenkang Dai, Guoqing Yang.

**Resources:** Rui Xie, Minghui Zhuang.

**Software:** Xiaojuan Chang, Guoqing Yang.

**Supervision:** Jie Yu, Liguo Zhu.

**Writing – original draft:** Wenkang Dai.

**Writing – review and editing:** Wenkang Dai, Jie Yu, Liguo Zhu.
